# Supplementary material for: Are carbon emissions trading and green financial instruments synergistic? -Comprehensive quantitative research based on content analysis
Source: PLoS One. 2024 Mar 7;19(3):e0298601. doi: 10.1371/journal.pone.0298601 (PMC10919601; doi:10.1371/journal.pone.0298601)
Supplement: S1 Data — (ZIP) [file pone.0298601.s001.zip › Data/地级市Z_F工作报告.zip!/║■─╧/╙└╓▌╩╨/└┤╫╘╣1⁄2╓┌║┼í╛┬φ┐╦╩2╛▌═°í┐_2018.docx]

各位代表:

现在,我代表市人民政府向大会报告工作,请予审议,并请各位政协委员提出意见。

一、2017年工作在克难奋进中取得新发展

2017年是我市发展史上极不平凡、卓有成效的一年。全市人民在市委的坚强领导下,认真学习贯彻党的十九大精神,紧紧围绕建设创新开放新高地、品质活力新永州,突出打好“六大战役”,着力抓好“四个提升”,有效克服经济下行压力和历史罕见洪涝灾害等困难挑战,保持了经济社会平稳健康发展。预计完成地区生产总值1750亿元,增长8%;规模工业增加值380亿元,增长7.5%;固定资产投资1870亿元,增长13%;社会消费品零售总额649亿元,增长10.8%;财政总收入168亿元,增长12%;城乡居民人均可支配收入分别达到26110元、12660元,分别增长8.7%、8.4%;全面小康实现程度提高到88.1%,同比提升4.5个百分点。

脱贫攻坚成效显著。把脱贫攻坚作为最大的政治任务和最重的民生工程,调集最优势的资源,汇聚最广泛的力量,实行最严格的军令状管理,推动脱贫攻坚取得决定性进展。预计全年减贫17.23万人,贫困村出列349个,宁远、双牌、江永3个省扶县可实现脱贫摘帽,全市贫困发生率由6.3%下降到3.3%。抓党建促脱贫攻坚、“三走访三签字”活动、中国社会扶贫网试点以及产业扶贫、金融扶贫、教育扶贫、健康扶贫等工作走在全省乃至全国前列。开工建设易地扶贫搬迁项目84个,安置建档立卡贫困户56541人。建成贫困村电商营运站点539个、光伏电站557个,116个村入选全省旅游扶贫示范村。投放扶贫小额贷款7.02亿元,贫困村金融服务站实现全覆盖。完成贫困劳动力转移就业1.29万人,贫困对象技能培训1.4万人。贫困学生义务教育完成率、贫困人口居民医保参保率均达到100%,救治救助贫困大病患者3.86万人次。贫困村基础设施和基本公共服务大幅改善。

产业培育势头良好。大力实施“园区升级战役”,全市工业园区完成基础设施投入108亿元,新增标准厂房103万平方米,新入园投资5000万元以上工业项目110个,竣工投产65个,新入统规模工业企业120家,新增首发上市和新三板挂牌企业3家,新认定高新技术企业30家。永州经开区申报国家级经开区通过国家相关部委审核,祁阳经开区总产值突破200亿元,道县、蓝山工业园新晋为百亿园区。编制修订农业园区规划150个,重点升级的14个农业园区投入资金23.9亿元,新引进农业项目20个、总投资172.1亿元。粮食、烤烟、油茶、蔬菜、水果、畜牧水产等大宗农产品生产稳中有升,新创农业标准化基地(场)60个,新增“三品一标”农产品130个、国家地理标志证明商标和产品5件,成功创建国家级出口食品农产品质量安全示范市。大力实施“旅游升温战役”,全市开工建设旅游重点项目63个,完成投资103.6亿元。获评全国森林旅游示范市,新增3A级景区6个,九嶷山舜帝陵入选国家级风景名胜区,零陵古城建设势头良好。成功举办纪念周敦颐诞辰1000周年、全国第四届草书展、第十一届阳明山“和”文化旅游节、湖南冬季乡村旅游节等重大节庆活动。全市接待国内外游客3400万人次,实现旅游综合收入200亿元,分别增长18.5%和22.7%。全年电商交易总额突破300亿元,新田获批全国电子商务进农村示范县。全市金融机构存、贷款余额分别达到2250亿元、1190亿元,分别增长17.5%、23.1%,新增贷款223.9亿元,新设5家村镇银行,光大银行入驻永州。房地产市场保持稳定,去库存效果明显。

城乡面貌持续改观。强力推进“城镇提质战役”十大工程建设,全市实施城镇提质重点项目212个,完成投资367.3亿元;市本级开工城建项目43个,完成投资137.2亿元,增长37.4%。“一宫两馆六中心”、湘江西岸片区道路改造、旧城及棚户区改造、城南大桥等一批重点项目加速推进,育才路提质改造、32条主干道路绿化提质及重点地段楼宇景观提档升级全面完成。国家卫生城市、国家森林城市创建成果巩固深化,国家历史文化名城保护力度加大,国家园林城市、全国文明城市创建工作全面展开。县城扩容提质和特色小镇建设来势较好,祁阳县、东安县芦洪市镇国家新型城镇化试点及回龙圩等国家、省级重点镇建设扎实推进,江华获批国家园林县城,宁远县湾井镇入选国家特色小镇。启动建设省、市美丽乡村示范村62个,双牌县坦田村入选国家人居环境整治示范村,江华井头湾村授牌为中国少数民族特色村寨。

基础设施日趋完善。深入实施“交通提速战役三年行动计划”,湘桂铁路永州地区扩能工程开工建设,永清广高铁列入湘粤两省合作备忘录,经停永州高铁增加至20对;零陵机场迁建获国家民航局批复,新开通永州至重庆、北京直飞航线;衡永高速公路前期工作进展顺利,S355、S356等重点干线公路和祁阳大道、冷东一级公路等潇湘城市群市政化快速通道建设加速推进,零双一级公路建成通车,中心城区BRT快速公交即将建成;完成各类农村公路建设2000余公里。农田、水利、电力、信息建设稳步推进,完成土地整治27.2万亩、高标准农田建设7.2万亩;涔天河水库扩建枢纽工程基本建成、移民工程通过国家验收、灌区工程进展顺利,毛俊水库开工建设;完成电网建设投资6.4亿元,神华国华永州电厂复工建设,一批大型风电、光伏项目建成并网发电;智慧永州“四中心”和城乡网格化信息系统上线运行,华为云计算中心、“宽带乡村”和“电信普遍服务试点”等项目进展良好,广播电视“村村响、户户通”工程全面完成。

生态环境整治有力。全面启动国家生态文明建设示范市创建工作,深入实施湘江保护和治理第二个“三年行动计划”,强力推进增绿、洁地、净水、治气四大工程,全面推行“双河长制”,全力抓好中央环保督察交办问题整改和信访件办理,有效解决了一大批历史治污难题。湘江出境水质保持Ⅱ类以上标准,中心城区饮用水源地水质达标率100%、空气质量优良率达到85.9%,全市畜禽养殖废弃物资源化利用率达到75%。完成人工造林28.2万亩,全省首个市级航空护林站投入使用。江华获评首批国家生态文明建设示范县,宁远成为全国首批农村生活垃圾分类和资源化利用示范县,道县列入全国畜禽粪污资源化利用重点县,江永永明河创建为国家湿地试点公园,祁阳太白峰获批国家森林公园、浯溪获批国家水利风景区,金洞获批全国森林康养基地试点。

改革开放深入推进。全面深化改革的“永州路径”持续拓展,简政放权、商事制度改革、农村综合改革、社会治理机制改革等多项改革走在全省前列。市政府工作部门再次精简行政权力44项,企业登记升级为“40证合一”,获批全省“互联网+政务服务”唯一试点城市,创新开展“五集中”审批服务,推动大众创业、万众创新活力持续迸发。全市新登记各类市场主体42056户、企业6631户,分别增长17.2%、21.2%。国家级“星创天地”达到3家,“院士工作站”实现零的突破。中信重工、三一重能、贵德光电、远大住工等一批行业领军企业成功落户,与央企省企对接成果丰硕。全年实际利用外资10.48亿美元、增长9.97%,内联引资265亿元、增长 15%;完成外贸进出口总额88亿元,增长30%。湖南(道县)农副产品集中验放场暨永州公路口岸、江华海关工作站开关运行。

民生事业稳步发展。实施“民生微实事建设年”活动,加大为民办实事力度,全市涉及民生的财政支出占比达到72%,省定10项重点民生实事全面完成,市定6284个民生微实事项目完成投资107.9亿元。新增城镇就业6.2万人、农村劳动力转移就业4.3万人。改造各类棚户区43668户、农村危房26927户。巩固提升农村饮水安全40万人。城乡低保清理提标,养老保险提标扩面,城乡居民医保制度整合全面完成。加快补齐社会事业短板,新建义务教育合格学校 177所、农村公办幼儿园 24所、城区学位5.2万个,高考二本以上上线人数增幅居全省第一。全社会研发经费增长25%以上,国家知识产权示范城市进入培育期,全国质量强市示范城市获准创建。文化惠民工程深入推进,群众文化体育活动和精神文明创建活动蓬勃开展,荣获全国群众体育先进单位。新建乡镇卫生院49个、行政村卫生室1073个、社区卫生服务中心9个,公立医院全部取消药品加成,计生工作转型发展。创建学校标准化食堂100家,群众食品安全满意度提高。

社会大局和谐稳定。着力加强社会治理,以网格为基本单元的“1+N”治理模式全面推行,村、社区(居委会)“两委”换届工作圆满完成。大力加强立体化、信息化、动态化社会治安防控体系建设,建成全国最大的人脸识别应用基地,建成全省第一个公安大数据实战平台。保持打击各类违法犯罪活动的高压态势,成功侦办“善心汇”等一批国字号、省字号重大案件,公安各项业务指标均居全省前列,全市社会治安公众评价全省第一,连续三年评为全省综治工作先进市。扎实开展 “信访积案清零”专项行动,切实解决群众合理诉求,信访形势明显好转。全力管控安全生产领域风险,事故起数和死亡人数连续10年“双下降”。

政府建设不断加强。坚决贯彻全面从严治党要求,狠抓从严治政。扎实推进“两学一做”学习教育常态化制度化,深入开展“解放思想,实干兴永”大讨论。切实加强法治政府建设,始终坚持依法行政,稳步推进政府立法工作。认真执行市人大及其常委会的决议、决定,自觉接受市人大及其常委会的法律监督、工作监督,主动接受市政协的民主监督和社会舆论监督。办理人大代表议案、建议299件、政协委员提案286件,宋家洲整治、修通并村断头路等一批重点议案、建议和提案办理成效明显。不断强化行政监察和审计监督,持之以恒纠“四风”治陋习反腐败,严肃查处了一批违规违纪违法案件。统筹经济建设和国防建设,国防动员、人民防空和国防后备力量建设稳步推进,军民融合深入发展。民族宗教、外事侨务、新闻出版、档案、史志、贸促、对台、气象、地震、老龄、慈善、残疾人、红十字会等工作齐头并进,工会、共青团、妇联等群团组织作用得到有效发挥。

各位代表,回首2017年的工作,我们深感成绩来之不易,倾注了全市人民共同的智慧、心血和汗水。一年来,因为有市委总揽全局、协调各方、科学指挥,团结带领干部群众积极应对经济下行压力,战胜历史罕见的特大洪涝灾害,在区域竞相发展态势中创新争先,为引领发展锻造了坚强核心;因为有市人大、市政协在参与中监督、在监督中支持、在支持中鼓劲,深入调研出主意,联系项目解难题,为加快发展营造了良好氛围;因为有驻永部队、武警消防官兵、民兵预备役及政法干警始终把群众生命财产安全放在首位,奋战在危难险重最前沿,为保障发展塑就了中流砥柱;因为有各民主党派、工商联、人民团体、无党派人士、离退休老同志以及其他社会各界人士情系永州,献计出力,为共谋发展凝聚了强大合力;因为有全市广大干部群众以改革发展稳定为己任,在各行各业辛勤劳作、创新创业、奉献付出,为支撑发展奠定了稳固基石。在此,我谨代表市人民政府,向大家表示衷心的感谢,并致以崇高的敬意!

各位代表,自信者当自醒,自醒者必自强。在总结工作的时候,我们既要肯定成绩,也要正视不足,清醒认识到当前制约永州发展的基础性、结构性、根本性问题。一是城市和集镇对经济发展的带动作用还不强。中心城区的集聚力、辐射力、承载力不强,功能不全和品质不高的问题同时存在,县城和集镇建设滞后于新型城镇化的要求,以城带乡的作用发挥不充分。二是创新和开放对经济发展的引领作用还不强。部分干部群众思想观念跟不上形势发展的需要,科技、管理、文化等方面创新能力较弱,开放平台建设与经济社会发展不相适应,开放型经济发展的规模和水平明显偏低。三是产业和园区对经济发展的支撑作用还不强。产业结构不适应经济转型升级的要求,工业经济规模不大,现代农业基础薄弱,现代服务业发展不足,特别是园区经济特色不鲜明,主导产业和产业链培育不够。四是环境和要素对经济发展的保障作用还不强。政务服务效率不高,少数人工作落实和执行不力,为政不勤不廉的现象仍然存在。资金、土地、人才等发展要素保障不到位。五是满足人民美好生活需要的条件还很不充分。城区学位严重不足,能上学的问题解决之后,上好学的问题仍然突出。城乡医疗服务体系还不完善,优质医疗资源非常紧缺。脱贫攻坚压力仍然很大。生态文明建设基础薄弱,部分环境突出问题一时难以解决。就业、增收的任务十分艰巨。一些涉及老百姓利益的信访问题还没有得到及时有效解决。不安定不稳定不确定的社会风险仍然不少。等等。

发展短板就是发展潜力,发展不足就是发展空间。上述问题,既是导致2017年部分目标任务没有完成的主要原因,也是当前和今后一段时期做好政府工作的主攻方向。对此,我们一定要坚持问题导向,强化问题意识,切实提高解决问题的能力。我们坚信,只要勇于面对、敢于担当,下定决心补短板,千方百计强弱项,就完全有信心、有能力、有办法应对前进道路上的各种困难和挑战。

二、奋力推动2018年工作迈上新台阶

2018年是全面贯彻落实党的十九大精神的开局之年,是改革开放40周年,是决胜全面建成小康社会、实施“十三五”规划承上启下的关键一年,更是我市在新的发展起点上融入新时代、开启新征程、展现新作为的起步之年。

新的一年,我们将面临经济下行压力持续加大、周边竞争态势日趋激烈、我国社会主要矛盾变化等新情况,必须做好科学应对的充分准备。同时又要看到,党的十九大描绘了决胜全面建成小康社会、夺取新时代中国特色社会主义伟大胜利的宏伟蓝图,必将激发和汇聚起645万永州儿女奋发图强、奋力前行的磅礴力量;随着国家“一带一路”、粤港澳大湾区建设等重大战略的全面实施,永州的区位、资源、生态、人文等优势将更为凸显;尤其是创新开放新高地、品质活力新永州建设的深入推进,给潇湘大地注入了蓬勃生机和巨大活力,永州发展的能量正在加速释放,必将迎来一个大有希望、大有作为的黄金期。我们一定要坚定信心、振奋精神,在新时代的新征程上勇立潮头、破浪向前。

根据市委部署,2018年全市经济社会发展的总体要求是:全面贯彻党的十九大精神,以习近平新时代中国特色社会主义思想为指导,加强党对经济工作的领导,坚持稳中求进的工作总基调,坚持新发展理念,紧扣社会主要矛盾的变化,按照高质量发展的要求,统筹推进“五位一体”总体布局,协调推进“四个全面”战略布局,坚持以推进供给侧结构性改革为主线,统筹推进稳增长、促改革、调结构、惠民生、防风险各项工作,大力实施“开放兴市产业强市”战略,积极拓展“六大战役”和“四个提升”,努力建设创新开放新高地、品质活力新永州。

主要预期目标是:地区生产总值增长8%以上,规模工业增加值增长8%以上,固定资产投资增长13%左右,社会消费品零售总额增长11.5%左右,进出口总额增长15%以上,财政总收入增长8%以上,城镇居民人均可支配收入增长8.5%以上,农村居民人均可支配收入增长9%以上,全面小康实现程度提升4个百分点以上,坚决完成环境保护、资源节约、安全生产等约束性指标。

实现明年发展目标,推动永州工作迈上新台阶,要着力从宏观层面把握以下几条:必须坚持以政治建设为统领。坚定不移在思想上政治上行动上同以习近平同志为核心的党中央保持高度一致,坚决、认真、全面贯彻执行中央、省委、市委的决策部署,切实把中央大政方针、省委要求部署落实到永州改革发展的具体实践,把市委的重大决策、重大安排贯穿于政府工作的方方面面。必须坚持以经济建设为中心。始终聚焦经济发展不快不优、综合实力不大不强的最大短板,更加自觉地把高质量发展作为第一要务,坚定不移地以新发展理念引领经济新常态、开辟发展新境界,努力走出一条既有较快增长速度、又有较高增长质量的追赶型发展路子。必须坚持以改革创新为动力。始终把改革创新作为破解当前各种难题、厚积未来发展能量的根本动力,突出以供给侧结构性改革为主线,以科技创新为引领,谋划推出一批具有重大牵引作用的改革创新举措,加快形成适应经济新常态的体制机制,着力塑造促进经济新发展的强大动能。必须坚持以民生优先为导向。坚定不移地践行以人民为中心的发展思想,始终把民生需求作为政府作决策、抓落实的基本取向,围绕不断满足人民群众日益增长的美好生活需要,持之以恒为群众办实事、做好事、解难事,切实用民生成效检验政府的执政理念、行政能力、工作绩效,不断提高人民群众对政府工作的满意度。必须坚持以铁的作风为保障。认真落实习近平总书记关于进一步纠正“四风”、加强作风建设的重要指示精神,以永远在路上的恒心和韧劲,全面推进各级政府作风建设,以埋头务实、精细严谨、优质高效、锲而不舍的过硬作风,推动政府各项工作扎实落地,促进永州各项事业持续发展。

(一)深入推进脱贫攻坚,奋力冲刺全面小康

坚决打赢脱贫攻坚战役。坚持真脱贫、脱真贫,一手抓已脱贫人口的稳定巩固,一手抓深度贫困人口的脱贫攻坚,全年实现减贫12万人以上,贫困村出列267个,新田、江华2个国扶县脱贫摘帽,贫困发生率下降2个百分点。继续深化“四跟四走”产业扶贫工作,切实抓好特色农业、电子商务、乡村文化旅游等产业项目,加快形成“一村一品、一户一业”的产业扶贫格局。切实抓好劳务协作脱贫,推行在贫困村建立“扶贫车间”,新增贫困劳动力转移就业8500人。加大金融扶贫力度,新增小额扶贫贷款5亿元,拓宽扶贫“特惠保”保障范围。加大贫困人口住房保障力度,完成易地扶贫搬迁2万人,优先抓好贫困人口危房改造。精准落实教育扶贫、健康扶贫政策,坚决不让一个贫困学生因贫失学、辍学,不让一个家庭因病致贫、因病返贫。加大涉农资金整合投入,加快补齐贫困村基础设施短板,提升医疗卫生、科技、文化、社会保障等公共服务水平。拿出最大财力,调动各方资源,激发贫困人口内生动力,集中力量攻克42个深度贫困村。激励引导民营企业、公民个人、社会组织参与脱贫攻坚,深入推进“万企帮万村”和“小微企业进村”行动,推动互联网+社会扶贫工作继续走在全国前列。加强扶贫领域项目资金管理,切实提高扶贫项目受益精准度和扶贫资金使用效益。

大力促进县域经济崛起。认真落实促进县域经济发展的政策措施,在做大做强市本级的同时,加大对县域经济发展的支持力度,不断增强县域发展活力和整体实力。着力加大对县区财源建设、产业发展和园区建设的支持力度,建立动态奖励机制,鼓励县区加大产业发展力度。支持在永金融机构网点向县域拓展,支持市县区融资平台、担保机构开展合作,支持县区向上争资金、争项目、争试点,重大项目优先列入市重点项目建设计划。坚持分类施策、错位发展,引导各县区优势优先发展、特色特殊发展,推动中心城区走在前列、经济强县率先发展、发展潜力县进位争先、重点生态功能县绿色崛起、民族特色县做优做强。优化县域经济发展量化考核评价机制,不简单搞“一刀切”“齐步走”,激励各县区主动作为、创先争优。科学合理划分市县区事权与支出责任,推动权力下放、权责一致。不断改善基层工作生活条件,加大乡村社区运转和人员待遇保障。统筹规范针对乡镇的评比表彰、示范创建等活动,未经批准不得对乡镇设置“一票否决”事项,切实为基层减负减压。

全面强化基础设施支撑。围绕建设“区域性综合交通枢纽城市”,以“两高两航三路一港”为重点,加快完善立体交通体系。扎实推进湘桂铁路永州地区扩能工程,积极推动和参与呼南高铁邵永客专前期工作,力争永清广高铁纳入《粤港澳大湾区城市群发展规划》和国家中长期铁路网调整规划;努力争取开工建设衡永高速公路,启动永郴高速、零道高速、永州至新宁高速公路前期工作;做好零陵机场迁建前期工作,新开通2至3条航线;启动湘江永州至衡阳千吨级航道改造工程,完成祁阳、冷水滩、零陵3个千吨级码头前期工作;加快潇湘城市群市政化快速连接道路建设,争取冷东、冷祁、S217(九嶷大道北出口)快速通道建成运行,上大(金)公路开工建设;实施以完善县区互通互联为目标的国省干线公路750公里,完成以通组公路为重点的各类农村公路建设改造5000公里,实施危桥改造74座;加快永州内陆港建设。抓好涔天河水库枢纽工程扫尾、灌区工程推进和毛俊水库建设三大“国字号”水利工程,加快一批在建中型水库建设步伐,力争零陵何仙观水库列入省水利工程PPP试点项目,打造中心城区“第二饮用应急水源”,完成1座大型水闸、26座病险水库除险加固、5个小农水项目县建设任务,推进高效节水灌溉、水土流失、中小河流治理、水电增效扩容和农村自来水工程。加快神华国华永州火电项目建设,开工建设总装机50万千瓦的风力发电、20兆瓦的光伏发电项目,抓好江华冯河110千伏输变电及蓝山大桥、双牌倪家洞等风电送出工程,协调推进各级电网建设改造,加快实施“气化永州”工程,力争永州至东安、双牌、道县天然气长输管线与衡阳至永州主管网同步开工建设。加快推进信息基础设施建设,完成“智慧永州”二期工程,抓好第三批“电信普遍服务试点”项目实施,实现所有行政村通光纤。

(二)深入推进产业强市,大力振兴实体经济

扎实抓好产业项目建设年活动。积极对接省里“百千万投资促进计划”和产业项目建设年活动,实施“五个10”产业项目建设工程,突出抓好10个重大产业项目、10个重大科技创新项目、10个重大产品创新项目,引进10个500强企业、10名重点科技创新人才,全力营造抓项目兴产业促发展的浓厚氛围。积极跟踪衔接,力争已申报的一批项目进入省里产业项目建设“五个100”笼子。

扎实抓好园区升级和工业提速。完成园区基础设施投入100亿元以上,新建标准厂房面积100万平方米以上,引进落户投资5000万元以上工业项目100个以上,建成投产60个以上,新增规模工业企业100家以上,新创1至2家省级高新技术产业开发区,抓好汽车制造及配套产业园、大数据产业园等特色园区建设。大力发展现代农业产业园,力争永州国家农业科技园创建为国家现代农业产业园,新创省级现代农业特色产业园示范园18个以上,实施配套升级14个以上。创新园区建设模式,主动与央企、行业龙头共建产业联盟和园中园。加速推进产城融合,不断完善园区功能配套,推动服务共享,促进工业园区城市化发展。深化园区人事、财税和投融资体制改革,探索建立“园政合一”管理模式,激发园区发展内生动力。实施新兴优势产业链和骨干龙头企业培育行动,着力打造先进装备制造、电子信息、生物医药、新能源新材料、节能环保、大数据等新兴产业集群,加快改造提升水泥建材、农产品加工、矿产品加工、轻纺制鞋等传统产业。整合财政专项资金,建立财源企业库,建立产业发展引导资金和企业增长奖励机制,加大对主导产业和优势骨干企业扶持力度。开展百户骨干企业精准帮扶活动和降成本专项行动,提高金融支持实体经济的精准度和实效性,健全中小企业融资担保体系,全年新增贷款200亿元以上,争取1至2家企业首发上市或新三板挂牌。

扎实抓好旅游升温和服务业提质。启动全市全域旅游发展总体规划编制工作,支持东安、宁远、江永创建国家全域旅游示范区、双牌创建“国际慢城”、九嶷山创建国家生态旅游示范区。推动国内知名旅游投资机构来永发展,加快零陵古城、永州华侨城、江华瑶族文化博览园、蓝山云冰山景区等重点项目建设,推进九嶷山创5A、周敦颐故里创4A工作,打造一批乡村旅游示范村、少数民族特色村镇、森林小镇和秀美村场。加强旅游配套设施建设,实施旅游整体营销工程,办好重点旅游文化节庆活动,推动大湘南和“湘粤桂城市圈”全域旅游联动发展,实现旅游总收入增长20%以上。加快万商红商贸城、潇湘汽车城、永州火车站物流园、粮食仓储物流产业园等重点商贸物流项目建设,引进、培育一批具有核心竞争力的大中型商贸物流企业,积极发展智慧物流,促进传统商贸物流业转型升级。举办好车展节、农博会,精心筹办一批新型展会。坚持“房子是用来住的”,引导房地产市场健康稳步发展,加快消化库存,建设一批适应市场需求的中高端小区。大力发展电子商务,实现电商交易总额突破400亿元,新创1个国家级电子商务进农村示范县。壮大金融服务业,力争新设立2家全功能县域支行,实现村镇银行全覆盖。

扎实抓好创新创业。深入实施创新驱动战略,实现全社会研发经费增长25%以上,新认定高新技术企业20家以上,实施省以上科技计划项目30项以上,争创国家或省级研发及创新平台2家以上。支持园区、企业与高等院校、科研机构、行业协会等共建双创基地,建设一批省级以上双创平台。全年新增市场主体4.2万个以上。抓好国家知识产权示范城市和全国质量强市示范城市创建工作,着力培育一批品牌产品、品牌企业、品牌工程。深入实施招才引智计划,完善人才引进的政策措施,加强本土人才培养,加强职工技能培训和素质提升,强化创新驱动发展的人才支撑。

(三)深入推进改革开放,加速集聚发展动能

突破重点改革。全面落实中央和省里确定的改革任务,更大力度抓好自主改革项目。深化“放管服”和商事制度改革,全面落实“双告知”、“双公示”和信用信息归集共享,全面推行“双随机、一公开”监管,抓好“互联网+政务服务”试点,办好网上政务大厅,推动审批服务从“一次办”、“马上办”到“网上办”。深化投融资和财税、金融体制改革,按照“有保有压”原则,在防范和化解财政、金融风险的同时,确保建设和民生资金需求。推动平台公司市场化转型发展,支持开展产业开发,加强城建类金融担保体系建设,破解城市建设融资瓶颈。进一步理顺国资监管体制,继续加大央企省企对接和成果有效落地。积极探索推行行政执法体制改革,增强行政执法效能,加快政府职能转变。

深化开放合作。大力实施开放兴市战略,全力打造对接东盟桥头堡,深度融入粤港澳大湾区,推动形成全面开放新格局。实施走进500强链接大产业行动,瞄准国内、世界和行业500强企业,量身定做招商项目,积极推动以商招商、园中园建设招商、基金招商,着力引进一批投资强度大、科技含量高、创新能力强的战略投资者,争取引进一个,带来一片,发展一群。充分发挥永州异地商会作用,深入开展引老乡回故乡建家乡活动,积极鼓励永商回乡创业。落实招商政策,优化招商服务,提高项目履约率、落地率、投产率。全年实际利用外资11.5亿美元以上,内联引资300亿元以上。实施构筑大通关提升大平台行动,拓展湖南(道县)农副产品集中验放场暨永州公路口岸功能,加快将湘南进出口食品(农副产品)检测中心打造成国家富硒农产品检测重点实验室,抓好江华海关工作站运行,推广国际贸易“单一窗口”。实施融入“新丝路”拓展大合作行动,持续推动外贸“破零倍增”,加快“永品出境”“永企出海”。加快外贸转型升级,培育壮大一批生产型外贸企业,大力发展总部经济,支持跨境电商企业做大做强。实现外贸进出口总额突破100亿元。

推动民营活市。认真落实国务院促进民间投资“26条”,推动国资、民资、外资全面对接合作。大力清理、督办、整改招商引资的违约失信问题,扎实开展招投标违规行为、乱收费乱罚款乱摊派及“雁过拔毛”式腐败等专项治理,着力优化营商环境。构建“亲”“清”新型政商关系,努力形成民营经济发展的“洼地”效应。

(四)深入推进城乡融合,着力打造美丽永州

推进城镇提质行动。加速提升中心城区首位度。以零冷两区联城为重点,以项目建设为支撑,加快做大做强中心城市。全年实施重点城建项目76个以上,完成投资125亿元以上。扎实抓好永州大道沿线开发,加快启动湘江风光带建设,全速推进滨江新城路网、“一宫两馆六中心”、二广高速永州东连接线、城南大道、潇湘大道、湘江两岸棚户区改造等一批重大项目,合理布局停车场、公厕、垃圾收集站等民生项目,完成城市公交与出租车改革,运行一期城市BRT。加大国家园林城市、全国文明城市等品牌创建力度,深入推进城市道路文明交通畅通提升工程。依托中心城区做大做强,加快推进潇湘城市群建设,突出抓好城市群内基础设施互联互通、功能配套,加快形成紧紧围绕中心城区的旅游圈、经济圈、生活圈。推进县城和小城镇扩容提质,着力完善路网、地下管网、“两房两棚”、“两供两治”等基础设施和民生工程,提升综合承载能力。持续抓好城镇品牌创建,新创一批国家、省级卫生县城、文明城市、园林县城。推动祁阳、道县“撤县设市”。探索小城镇建设融资途径,加快特色村镇建设,开展特色小镇创建活动,新建市级美丽特色小镇10个以上。深化城镇管理体制改革,加快完善各级城镇规划体系,严格规划实施与管理,加大控违拆违力度,加快智慧城镇建设,提升精细化管理水平。

实施乡村振兴战略。科学制定全市乡村振兴战略规划,选准并实施一批乡村振兴试点,积极探索乡村振兴的永州路径。深入推进农业供给侧结构性改革,坚持质量兴农、绿色兴农,推动农业发展由增产导向转变为提质导向。大力建设以精细农业、智慧农业为特色的优质农副产品供应基地,突出抓好粮食、生猪、油茶、柑橘、蘑菇、茶叶、生姜、芋头等8大优势产业基地建设。加速推进农业标准化建设,新建市级以上农业标准化示范基地(场)60个以上,新增1个国家级、2个省级农产品质量安全县。加强农业品牌建设,注重国家地理标志产品开发,抓好“三品一标”农产品认证,对优势农产品实行“全市一品”。积极培育新型农业经营主体,大力支持骨干龙头企业上市。深化农村综合改革,全面完成农村土地承包经营权登记颁证,积极探索“三权分置”有效实现形式,推进农村土地规范有序流转。全面开展农村集体产权制度改革试点工作,每个县区启动完成2个村的试点任务。加快农村金融保险创新,实现县区开展农村土地经营权抵押贷款全覆盖,积极引导工商资本进农村。深入实施“四清四化”和“五改”行动,大力推进农村“厕所革命”和集贸市场建设改造,抓实农村环境连片整治和农村清洁工程,实现所有行政村配备保洁员,农村垃圾集中收运率达到100%、生活垃圾集中处理率达到90%。新启动建设省、市美丽乡村创建村60个,推进环境整治重点村450个,争创全面小康示范村100个。设立乡镇规划委员会,加强农村建设规划管理。抓好第三次全国土地调查工作。

厚植生态文明优势。围绕创建国家生态文明建设示范市,深入实施“强规、增绿、洁地、净水、治气”工程,营造天蓝、地绿、水净的美好家园。强规方面,全面完成市县区创建国家生态文明建设示范市(县)的规划编制工作,制定出台生态文明建设目标评价考核办法。增绿方面,启动大规模国土绿化行动,抓好乡村“五边”造林和城市“六个绿色”创建,完成人工造林27万亩。洁地方面,全面实施“土十条”,加强土壤污染防治与修复,严格危险废物环境监管,污染地块安全利用率达到90%。净水方面,深入实施“水十条”,压实市县乡村四级河长责任,推进湘江保护与治理第二个“三年行动计划”,抓好湘江两岸退耕还林还湿试点。支持县区开展省级湿地公园试点申报,打造1至2个高标准、高品质的国家湿地公园。加强城市黑臭水体治理,实现县级以上集中式饮用水水源地水质达到Ⅱ类以上标准,3个国家考核断面达标率100%。治气方面,加快空气质量达标城市创建,强化建筑扬尘、餐饮油烟、燃煤锅炉、重点工业污染源等监管,确保中心城区空气质量优良率达到85%以上,全市大气质量综合指数在全省保三争二。

(五)深入推进民生事业,不断提升群众福祉

着力办好10件民生实事。1、深入实施“学位建设三年行动计划”,2018年基本消除66人以上超大班额;2、将城乡居民医保财政补助标准提高到年人均480元;3、救治救助150名无责任主体的农民工尘肺病患者;4、继续提高退休人员基本养老金和城乡居民基础养老标准;5、确保每千名老年人口养老床位数达到32张;6、实施棚户区改造2.6万套、农村危房改造1万户;7、困难残疾人生活补贴和重度残疾人护理补贴发放标准不低于每人每月50元;8、帮助240名0至6岁脑瘫儿童实施抢救性康复;9、为1400名智力、精神和重度肢体残疾人开展集中托养服务;10、实施主城区特别是老城区地下排水排污系统建设攻坚工程,加快改善居民生活环境。

认真办好人民满意教育。推进城乡义务教育一体化,深入实施“四名工程”,加快普及高中教育,争取1至2所示范性高中进入全省名校第一方阵。统筹发展高等教育、职业教育、特殊教育,支持发展民办教育,加快永州高等师范专科学校建设和市委党校搬迁,组建永州职业教育集团。下大力解决中小学生“课外负担重”“择校热”等突出问题,解决好婴幼儿照护和儿童早期教育服务问题,努力让每一个孩子都能享有公平而有质量的教育。

加快完善社会保障体系。以充分就业为目标,着力提高就业质量,确保新增城镇就业5.7万人、农村劳动力转移就业4万人,城镇登记失业率控制在4.5%以内。切实维护劳动者合法权益,着力治理拖欠克扣农民工工资问题。完善“五险”统一征缴制度。深化机关事业单位养老保险制度改革。创新医保支付方式,推广医保异地结算,提高医保基金使用成效。巩固农村低保标准和扶贫标准“两线合一”成果,提高城乡低保精准度。健全社会救助制度,加快慈善事业发展,关心关爱农村留守儿童、困境儿童、孤寡老人、残疾人和流浪乞讨人员。

扎实推进健康永州建设。推动医改向纵深发展,加快“分级诊疗”实施,加大全科医生培养,巩固药品零差率销售改革成果,严控医疗费用不合理增长。扎实抓好中心城区医疗卫生“四中心”建设,推进县级医院、基层医疗机构和公共卫生服务机构标准化建设。健全重大疾病防控工作机制,优化公共卫生服务。传承发展中医药事业。鼓励社会力量发展健康产业。推进健康、养生、养老与旅游、体育相融合。加强出生缺陷综合防治,提高出生人口素质。加大食品药品监管力度,新创学校标准化食堂200家。

大力促进文化繁荣发展。积极创建国家公共文化服务体系示范区,着力抓好市文化艺术中心、博物馆、图书馆、工人文化宫等重点文化设施项目建设,推进公共文化服务基本化、均等化、便利化。实施文化艺术名家扶持工程,培育一支高水平的文艺创作人才队伍。大力倡导全民阅读,积极建设“书香永州”。加强历史文化名城、名镇、名街、名村、名居等文化遗产和非物质文化遗产保护,支持女书申报世界记忆名录和世界文化遗产,打造中国书法名城。成立文旅投资公司,促进文旅投入与城建投入相结合,引进大品牌、新业态,整合开发文旅资源,做大做强文化产业,培育一批文化龙头企业。加强公民思想道德建设,强化网络空间治理,净化网络环境。

统筹发展其他各项事业。积极发展老龄事业,扎实做好老干部工作。加快推进《永州市志》编修,全面完成部门志编撰。认真做好民族宗教、外事侨务、统计、档案、保密、气象、地震、人防、水文等工作。充分发挥工会、共青团、妇联等群团组织作用。大力支持国防和军队改革,推进军民融合深度发展,加强国防后备力量建设,做好军转干部和退役士兵接收安置工作。

(六)深入推进平安创建,努力促进社会和谐

推进社会治理创新。进一步完善网格化服务管理,构建管理功能完善、参与主体多元的网格化服务管理体系。有效整合部门资源,推进综治中心一体化建设、实体化运作。大力实施“雪亮工程”,建立市、县(区)、乡镇(街道)、村(社区)四级联网的视频监控图像信息系统。建好用好省市县际公安检查站,推进乡镇和居民小区治安星级评比,推动群防群治各项措施落地落实。加快农村综合服务平台建设,实现乡镇、村居基层服务平台全覆盖。

深化平安永州建设。牢固树立总体国家安全观,全面加强反暴恐工作和国家安全工作,依法严厉打击黑恶势力、涉爆涉枪、“两抢一盗”、黄赌毒、传销、走私、电信诈骗、非法集资等各类违法犯罪,持续开展集中整治“三强三堵”、治安失控村、“村霸”和宗族恶势力等专项行动。健全完善矛盾纠纷多元化解机制,积极稳妥处置各类群体性事件。扎实推进“平安创建”,争创一批省级、市级平安示范单位。深入开展“信访法治化建设年”活动,大力推进“互联网+信访”,加强“四无乡镇(街道)”、“三无村(社区)”创建,强化源头治理。切实加强防灾救灾减灾能力建设,进一步完善应急管理体系,提高突发事件防范控制、应急响应、有效处置能力。

强化安全生产工作。坚守发展决不能以牺牲安全为代价这条不可逾越的红线。压实党委政府领导责任、部门监管责任、企业主体责任,健全公共安全体系,大力推进安全生产事业改革发展,全面落实风险隐患防治“一单四制”制度,严格实施“黄牌警告”“一票否决”制度,坚决遏制较大及以上安全事故发生,切实保障人民群众生命财产安全。

三、用更高更严标准塑造政府新形象

开启新征程,担负新使命,全市各级政府必须把自身建设摆在更加突出的位置,坚持以能力建设为引领,努力打造一个讲政治、敢担当、有作为、守清廉的人民满意政府。

提升政治能力,打造忠诚政府。突出政治建设在政府自身建设中的统领地位,将政治能力摆在干部能力的首要位置。全市政府各级干部特别是领导干部要牢固树立“四个意识”,坚决维护以习近平同志为核心的党中央权威和集中统一领导,扎实开展“不忘初心、牢记使命”主题教育,坚决按照省委、省政府和市委的工作要求,推动政府各项工作规范、高效、有序运转。坚决执行人大及其常委会的决议、决定,认真办理人大代表议案、建议和政协委员提案,自觉接受人大的法律监督、工作监督和政协的民主监督,虚心听取政协委员、民主党派、工商联、无党派人士和人民团体的意见。切实强化法治思维,把政府工作全面纳入法治化轨道,努力建设法治政府,统筹推进科学立法、严格执法、公正司法、全民守法。严格落实重大行政决策法定程序,认真执行民主集中制,严格按程序办事、按规则办事、按集体意志办事,提高科学决策、民主决策、依法决策水平。

提升创新能力,打造活力政府。建设创新开放新高地、品质活力新永州,关键在于用创新引领开放、引领发展。要把创新作为推动政府工作的活力源泉,发扬敢闯敢试、敢为人先的精神,用创新的思维、改革的意识、破局的办法来解决项目、土地、资金、民生等各类难题,大力推进思路创新、管理创新、政策创新。要以解放思想推动观念转变,破除工作惯性,努力从固有的思维定势中走出来,以思想认识的新飞跃打开工作的新局面。要把学习作为一种政治责任、一种精神追求、一种生活态度、一种工作需要,通过学习不断增强改革创新的能力,克服本领不足、本领恐慌、本领落后的问题。当前最为紧要的,就是要真正学懂、弄通、悟透党的十九大精神,坚持在融会贯通、学以致用上下功夫,增强推动高质量发展和建设现代化经济体系的能力。

提升执行能力,打造实干政府。工作都是干出来的、拼出来的、逼出来的。永州发展的光明前景和美好未来,要靠我们用自己的双手去创造。要大力弘扬和践行不知疲倦、不甘平庸、不断进取的奋斗精神,以不回避、不遮掩、不畏难的态度,一级做给一级看,一级带着一级干,用各级干部的辛苦指数换取人民群众的幸福指数。要深入改进作风,多到基层去,多到一线去,多到现场去,少讲话,少开会,少发文,力戒形式主义和官僚主义,集中更多的时间和精力解决实际问题,推动工作落实。对部署了的工作、决定了的事情,要盯住不放、立马就干,一杆子到底,真正做到踏石留印、抓铁有痕,力争做一件,成一件。坚决整治表态多调门高、行动少落实差等突出问题。宽容干部在工作中特别是改革创新中的失误,旗帜鲜明地为敢于担当者担当,为敢于负责者负责,真正让想干事的有机会、能干事的有舞台、干成事的有地位。

提升自律能力,打造廉洁政府。从政人员必须存敬畏、知戒惧、守底线,时刻把纪律和规矩挺在前面,严格落实党风廉政建设主体责任和“一岗双责”,严格遵守廉洁从政各项规定,守住个人干净的政治形象。深入贯彻中央八项规定实施细则,坚决防止“四风”问题回潮反弹,严控“三公”消费,把每一分钱都花在刀刃上,用政府的紧日子换取群众和企业的好日子。严厉查处各类违规违纪违法案件,坚决纠正损害群众利益的不正之风,加大对重点对象、重点部门、重要岗位、重点领域的监督力度,加大对财政资金、重大项目的审计力度,决不跟腐败分子搅到一起,决不给腐败分子可乘之机,决不容腐败分子藏身之地,把各级政府反腐败斗争进行到底,努力实现干部清正、政府清廉、政治清明、社会清新。

各位代表,新时代赋予永州发展新机遇,新使命呼唤永州人民新作为。让我们紧密团结在以习近平同志为核心的党中央周围,在市委的坚强领导下,不忘初心、牢记使命,锐意进取、埋头苦干,以永不懈怠的精神状态和一往无前的奋斗姿态,坚决打赢脱贫攻坚战役,决胜全面建成小康社会,奋力谱写加快建设创新开放新高地、品质活力新永州的崭新篇章!
